# Oral vaccination with *Trichinella spiralis* DNase II DNA vaccine delivered by attenuated *Salmonella* induces a protective immunity in BALB/c mice

**DOI:** 10.1186/s13567-018-0614-y

**Published:** 2018-12-05

**Authors:** Xin Qi, Yue Han, Peng Jiang, Xin Yue, Hua Nan Ren, Ge Ge Sun, Shao Rong Long, Chuan Yu, Xiang Chao Cheng, Jing Cui, Zhong Quan Wang

**Affiliations:** 1 Department of Parasitology, Medical College, Zhengzhou University, Zhengzhou, 450052 China; 2 Key Lab of Animal Disease and Public Health, College of Animal Science and Technology, Henan University of Science and Technology, Luoyang, 471003 China

## Abstract

Trichinellosis is one of the most serious foodborne parasitic zoonosis with worldwide distribution, and it is necessary to develop a vaccine to interrupt transmission from animals to humans. *Trichinella spiralis* adult-specific DNase II-1 (TsDNase II) were identified by immunoproteomics in surface or excretory/secretory proteins of adult worms (AW) and intestinal infective larvae (IIL). The aim of this study was to investigate the systemic, mucosal responses and immune protection elicited by oral vaccination with TsDNase II DNA vaccine delivered by attenuated *Salmonella typhimurium* strain⊿cyaSL1344. Oral vaccination with TsDNase II DNA vaccine triggered an obvious mucosal sIgA response and a systemic IgG response in mice, and IgG1 was predominant. Th1 (IFN-γ) and Th2 (IL-4, 10) cytokines were distinctly increased in the spleen and mesenteric lymph node (MLN) cells of vaccinated mice. An indirect immunofluorescent test revealed that native TsDNase II is present at the cuticle of this nematode after the 2^nd^ molting, further confirming that TsDNase II is adult-specific and expressed at AW and pre-adult stages. Oral immunization of mice with TsDNase II exhibited a 53.85% reduction in AW and a 59.26% reduction in ML after larval challenge. The in vitro NBL production of adult females from TsDNase II-vaccinated mice was also reduced in comparison with pcDNA3.1 or the PBS control group (*P* < 0.01). Our results show that oral immunization of mice with TsDNase II produced an intestinal and systematic concurrent Th1/Th2 immune response, and a significant immune protection against challenge.

**Electronic supplementary material:**

The online version of this article (10.1186/s13567-018-0614-y) contains supplementary material, which is available to authorized users.

## Introduction

Trichinellosis, one of the important zoonotic parasitic diseases worldwide, principally results from eating raw or semi-cooked meat containing *Trichinella* larvae [1]. *Trichinella* infection in humans is mainly caused by the species *Trichinella spiralis*, and domestic pork and pork products are the major infectious source [2–4]. China is one of the few countries with the highest number of trichinellosis patients, and there were 15 human trichinellosis outbreaks and four deaths were reported from 2004 to 2009 [5]. Trichinellosis is a serious threat on human health, and it also has a great impact on meat production and food safety [6, 7]. Since trichinellosis is transmitted from domestic or wild animals to humans by ingestion of contaminated meat, it is necessary to develop a vaccine to interrupt the transmission from animals to humans, and a preventive anti-*Trichinella* vaccine would make a substantial contribution to the control and elimination of trichinellosis [8–10].

After the encapsulated muscle larvae (ML) are liberated from contaminated meat in the host’s stomach, the ML migrate to the intestine and are activated into intestinal infective larvae (IIL), which invade the intestinal columnar epithelium and develop into adult worms (AW) after four moltings. From the beginning of about 5 days post-infection (dpi), female adults give birth to newborn larvae (NBL) that penetrate into the intestinal mucosa and are carried to the whole body via blood circulation. The NBL invade and encapsulate in the host’s skeletal muscle to complete its life cycle [11]. Therefore, the intestinal mucosa is the primary interaction place of the nematode with the host and the first natural barrier for combating *Trichinella* infection. The local intestinal mucosal immune response is important for immune protection against enteral *Trichinella* infection [12–15].

The AW is an important stage during the *T. spiralis* lifecycle. Vaccination with AW crude antigens provides a highly significant protection with an 89% and 80% reduction of AW and ML; vaccinated mice exhibit an accelerated expulsion of intestinal AW, a reduction in female fecundity and decreased ML burden [16]. In our previous studies, *T. spiralis* adult-specific DNase II-1 (TsDNase II, GenBank: AAY32316.1) were identified from IIL surface proteins and AW excretory/secretory (ES) proteins by immunoproteomics with early infection sera [17–20]. The full-length TsDNase II cDNA sequence was 1221 bp and the predicted ORF (1–1044 bp) encoded 347 amino acids. The predicted MW of TsDNase II is 38.06 kDa with a pI of 8.85. The recombinant TsDNase II protein (rTsDNase II) was expressed in *E. coli* in our laboratory. The mice subcutaneously vaccinated with rTsDNase II elicited a significantly high level of serum anti-rTsDNase II IgG, and exhibited a 40.36% intestinal AW reduction and a 50.43% ML reduction after larval challenge [21].

In this study, the plasmid pcDNA3.1/TsDNase II was constructed and delivered by an attenuated *Salmonella typhimurium* strain⊿ cyaSL1344 as a DNA vaccine. The systemic, mucosal responses and immune protective efficacy produced by oral vaccination with this TsDNase II DNA vaccine were observed in BALB/c mice.

## Materials and methods

### Parasite and animals

The *Trichinella* species used in our study was *T. spiralis* (ISS534), which was collected from an infected domestic pig in central China. This species was kept by passage in mice. Female BALB/c mice, 6 weeks old, were obtained from the Henan Provincial Laboratory Animal Center.

### Collection of worms and ES protein preparation

The ML at 42 dpi were collected by artificial digestion of infected mouse carcass using 0.33% pepsin and 1% HCl [22, 23]. The IIL was obtained from infected mouse intestines at 2, 4, 6, 12, 15, 18 and 24 hours post-infection (hpi) [17]. AW were recovered from intestines at 3 and 5 dpi. The NBL were harvested from 6 days old pregnant females cultured for 24 h in RPMI-1640 medium [24, 25]. AW ES proteins were prepared as described [26].

### Preparation of recombinant TsDNase II and anti-rTsDNase II serum

Full-length cDNA encoding TsDNase II was cloned into the pQE-80L, the pQE-80L/TsDNase II was transformed into *E. coli* DH5α [27]. The rTsDNase II was expressed under induction with 1 mM IPTG and was purified by Ni-affinity chromatography in our department [21, 28].

Ten mice were subcutaneously immunized with 20 μg of the rTsDNase II emulsified with complete Freund’s adjuvant, and boosted two times by the same dose of rTsDNase II with incomplete Freund’s adjuvant at a 10-day interval [30, 31]. Anti-rTsDNase II serum were collected at day 10 following the last immunization, and pre-immune normal serum was used as the negative control [21].

### Attenuated *Salmonella*

The attenuated *S. typhimurium* ⊿ cyaSL1344 strain with a mutation of adenylate cyclase gene was prepared by the Key Laboratory of Animal Disease and Public Health, Henan University of Science and Technology. The bacterium was served as the live carrier of the pcDNA3.1-TsDNase II [29].

### Plasmid construction and transformation

Complete TsDNase II cDNA sequence (GenBank: AY963695.1) was synthesized (Genewiz, Suzhou, China), then it was cloned into the pcDNA3.1 (Invitrogen, Carlsbad, USA) using BamHI and XhoI sites. To prepare the competent cells, attenuated *S. typhimurium* strain ⊿ cyaSL1344 was cultured at 37 °C overnight [29]. After being centrifuged, the pellet was washed three times in PBS (pH 7.2) and resuspended in PBS. The pcDNA3.1-TsDNase II and pcDNA3.1 were subsequently electroporated into the bacteria (Gene Pulser Xcell, Bio-Rad, Hercules, CA, USA). A positive transformant was selected on LB agar with 50 μg/mL ampicillin, then identified by PCR amplification and restriction enzyme digestion. The⊿cyaSL1344/pcDNA3.1-TsDNase II and ⊿cyaSL1344/pcDNA3.1 were respectively used as oral TsDNase II DNA vaccine and control.

### Detection of in vitro expression of TsDNase II gene by RT-PCR and indirect immunofluorescent test (IIFT)

Baby hamster kidney cells (BHK-21) were cultivated in plate (NEST, Wuxi, China) in RPMI-1640 containing 5% FBS without antibiotics [30, 31], then the recombinant pcDNA3.1-TsDNase II was transfected into the BHK-21 using Lipofectamine 2000 (Invitrogen, USA). Total RNA was extracted from transfected cells using Trizol (Invitrogen, USA), then the cDNA was obtained using AMV reverse transcriptase (Promega, USA). The TsDNase II transcription in BHK-21 was measured by RT-PCR using the primers (5′-GATTACCAATGCAAAGAACA-3′; 5′-TTAGGTGCATCCATCCAAGT-3′). Expression of TsDNase II was observed using IIFT [32, 33]. Briefly, BHK-21 was fixed with cold acetone at room temperature for 20 min. After washing, the cells were permeabilized using 1% TritonX-100 for 10 min, subsequently probed by 1:10 dilution of anti-rTsDNase II serum at 37 °C for 1 h. After being washed again, the cells were stained at 37 °C for 1 h by 1:100 dilutions of anti-mouse IgG-FITC conjugate (Santa Cruz, USA), then observed by fluorescence microscopy (Olympus, Japan).

### A vaccination project of oral TsDNase II vaccine and sample collection

One hundred and twenty BALB/c mice were divided into 3 groups with 40 animals per group, the vaccine group was orally inoculated with 1 × 10^8^⊿cyaSL1344/pcDNA3.1-TsDNase II. The other two groups received ⊿cyaSL1344/pcDNA3.1 or PBS alone. Vaccination was given three times at a 10-day interval. One hundred microliters of 10% NaHCO_3_ were administrated by gavage to all mice to neutralize gastric acids 30 min before oral inoculation [34, 35]. At 10 days after each immunization, 5 mice from each group were euthanized, and serum, spleen, mesenteric lymph nodes (MLNs) and intestinal lavage fluids were gathered to determine the levels of humoral and cellular responses to the TsDNase II immunization.

### The in vivo expression of TsDNase II detected using RT-PCR and IIFT

TsDNase II transcription in MLN and spleen tissues of vaccinated mice was measured using RT-PCR at 1 week after the first immunization. Total RNA were extracted from mouse MLN and spleen by Trizol (Invitrogen, USA). Mouse β-actin of MLN and spleen was also amplified as an internal control. The PCR product was analyzed on 1% agarose gels [36]. To determine the expression of TsDNase II, 5 μm cryosections of MLN and spleens were cut using a microtome. Tissue sections were fixed with cold acetone and blocked for 1 h at 37 °C in 5% normal goat serum. After washing, the sections were probed at 4 °C overnight using a 1:10 dilution of anti-rTsDNase II serum, then they were stained with anti-mouse IgG-FITC conjugate (1:100), and were finally observed under fluorescence microscopy (Olympus, Japan) [37, 38].

### Determination of specific antibody response by ELISA

Serum specific IgG, IgG1 and IgG2a against TsDNase II in vaccinated mice were assayed by ELISA using rTsDNase II [36, 39]. Briefly, the plate (Nunc, Roskilde, Denmark) was coated at 4 °C overnight with 1 μg/mL rTsDNase II, and blocked at 37 °C for 1 h using 5% nonfat milk. Immune serum (1:100) was added and incubated at 37 °C for 1 h. HRP-conjugated anti-mouse IgG, IgG1 or IgG2a (1:5000; Southern Biotechnology, USA) were added and incubated at 37 °C for 1 h. The coloration was developed by incubation with o-phenylenediamine dihydrochloride (OPD; Sigma) plus 30% H_2_O_2_, and terminated by 2 M H_2_SO_4_. Optical density (OD) at 490 nm was measured using a microplate reader (Tecan, Schweiz, AG, Switzerland), and serum samples were performed in duplicate [33].

### Determination of total and specific secretory IgA against TsDNase II

Total or TsDNase II-specific secretory IgA (sIgA) was assayed in intestinal washings of vaccinated mice [29]. To collect intestinal mucosal washings, 20 cm of small intestine was cut and the interior was rinsed with cold PBS containing a protease inhibitor cocktail (Sangon Biotech, China). The washing was centrifuged at 1000 *g* for 10 min, and total intestinal sIgA in supernatant was measured by sandwich ELISA with rabbit anti-mouse IgA (Abcam, UK) as the capturing antibody, and goat anti-mouse IgA-HRP conjugate as the detecting antibody [40]. TsDNase II-specific sIgA was determined using indirect ELISA with 1.0 μg/mL rTsDNase II or 2.5 μg/mL AW ES proteins as coating antigens. Optical density (OD) at 490 nm was assayed using a microplate reader (Tecan) [41].

### Recognition of the natural TsDNase II in different worm stages by IIFT

Recognition of natural TsDNase II on the cuticle and internal organs of various *T. spiralis* stage worms was assayed by IIFT with anti-TsDNase II immune serum and TsDNase II-specific sIgA from mice immunized orally with TsDNase II DNA vaccine [42, 43]. Whole worms and 3 μm sections of 3-day adult worms were fixed in acetone and blocked with 5% goat serum at 37 °C for 1 h. Worms and sections were probed at 37 °C for 1 h with a 1:10 dilution of immune serums or intestinal washings obtained 1 week after the 3^rd^ vaccination. Subsequently, they were incubated using anti-mouse IgG- or IgA-FITC conjugate (1:100; Santa Cruz Biotechnology, USA), and observed by fluorescence microscopy (Olympus, Japan) after being washed [44, 45].

### Cytokine determination

To determine cellular immune responses, spleen and MLN were collected from vaccinated mice at a 10-day interval after vaccination. Splenocytes were treated with 0.15 M NH_4_Cl, 20 mM Tris (pH 7.4) for 10 min at 4 °C to lyse erythrocytes, then washed twice in RPMI-1640 medium and the cell pellets were collected after centrifugation at 300 *g* for 10 min [14]. Spleen and MLN cell density was modulated to 2 × 10^6^ cells/mL in complete RPMI-1640 with 10% fetal bovine serum (FBS), penicillin (100 U/mL) and streptomycin (100 μg/mL). These cells were stimulated at 37 °C in 5% CO_2_ with 4 μg/mL of rTsDNase II for 72 h. The supernatant was harvested and cytokines (IFN-γ, IL-4, and IL-10) were examined using sandwich ELISA [46, 47]. Cytokine concentration was shown as picograms per milliliter (pg/mL).

### Challenge experiment

To evaluate the immune protection efficacy, 60 mice (20 animals per group) were inoculated orally with 300 ML at 10 days after the last vaccination. Ten mice of each group were sacrificed at 5 days pi and intestinal AW were harvested. ML were recovered and numbered from the remaining ten mice at 42 dpi [48]. The immune protection was estimated on the basis of the quantity of AW or larvae per gram (LPG) of muscle obtained from vaccinated mice relative to those from PBS control mice [9, 49]. The fecundity of adult females was determined according to the in vitro NBL production of each female adult in 72 h [50].

### Statistical analysis

The data were analyzed using SPSS version 17.0 software. One-way ANOVA (LSD test) was performed to analyze the intragroup or intergroup differences of serum anti-TsDNase II IgG level, total sIgA, TsDNase II-specific sIgA, cytokines, worm burden and NBL production. The Student’s *t* test was used to analyze the difference between serum IgG1 and IgG2a level on days 20 and 30 after the first vaccination. All data of OD values, cytokine production, and worm recovery are shown as the mean ± standard deviation (SD). *P *< 0.05 was justified as statistically significant.

## Results

### The in vitro expression of TsDNase II gene

**Figure 1:**
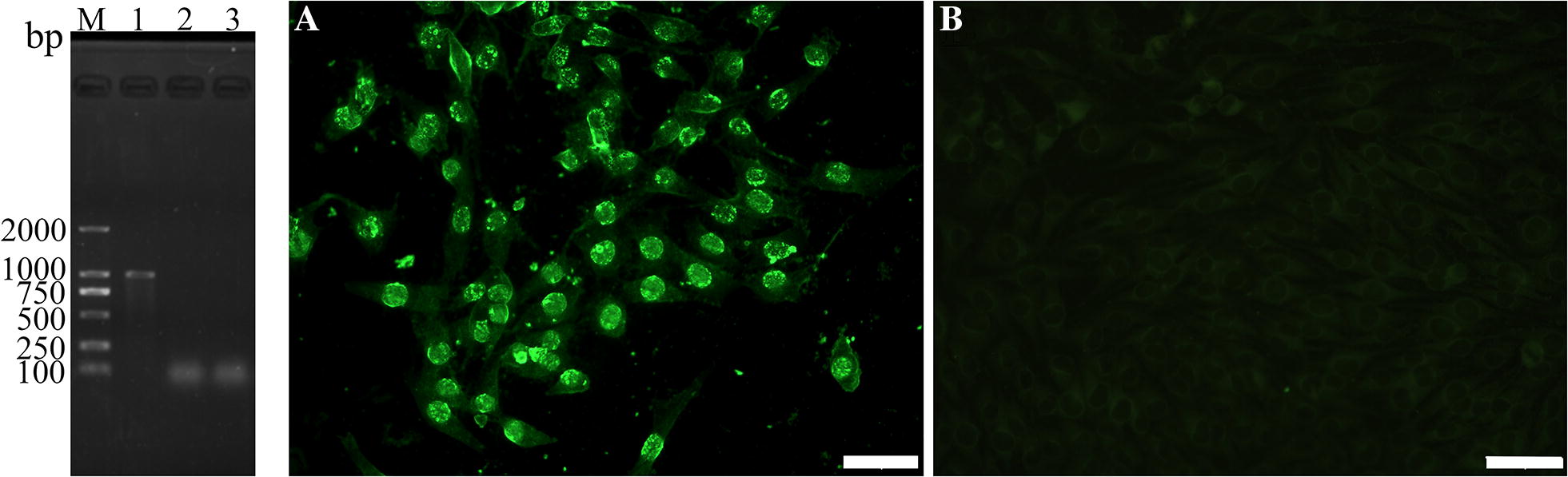
**The in vitro transcription and expression of TsDNase II in BHK-21 cells.** Left: TsDNase II transcription in BHK-21 cells was analyzed using RT-PCR. Lane M: DNA ladder; Lane 1: pcDNA3.1-TsDNase II transfected cells; Lane 2: only pcDNA3.1 transfected cells; Lane 3: ddH_2_O negative control. Right: TsDNase II expression in BHK-21 cells was detected by IITF with anti-rTsDNase II serum.** A** pcDNA3.1-TsDNase II transfected BHK-21 cells;** B** alone pcDNA3.1transfected BHK-21 cells. The scale: 50 μm.

Transcription of TsDNase II in BHK-21 cells was assayed using RT-PCR. The PCR product was stained using ethidium bromide on 1% agarose gels. The amplified TsDNase II fragments were detected in the cells transfected with pcDNA3.1-TsDNase II, but not in pcDNA3.1-transfected cells. Expression of TsDNase II in BHK-21 was detected by IIFT. By using anti-rTsDNase II serum, we detected a green fluorescence staining in pcDNA3.1-TsDNase II transfected cells, but no positive staining was detected in pcDNA3.1 control cells (Figure 1).

### The in vivo expression of TsDNase II gene

**Figure 2:**
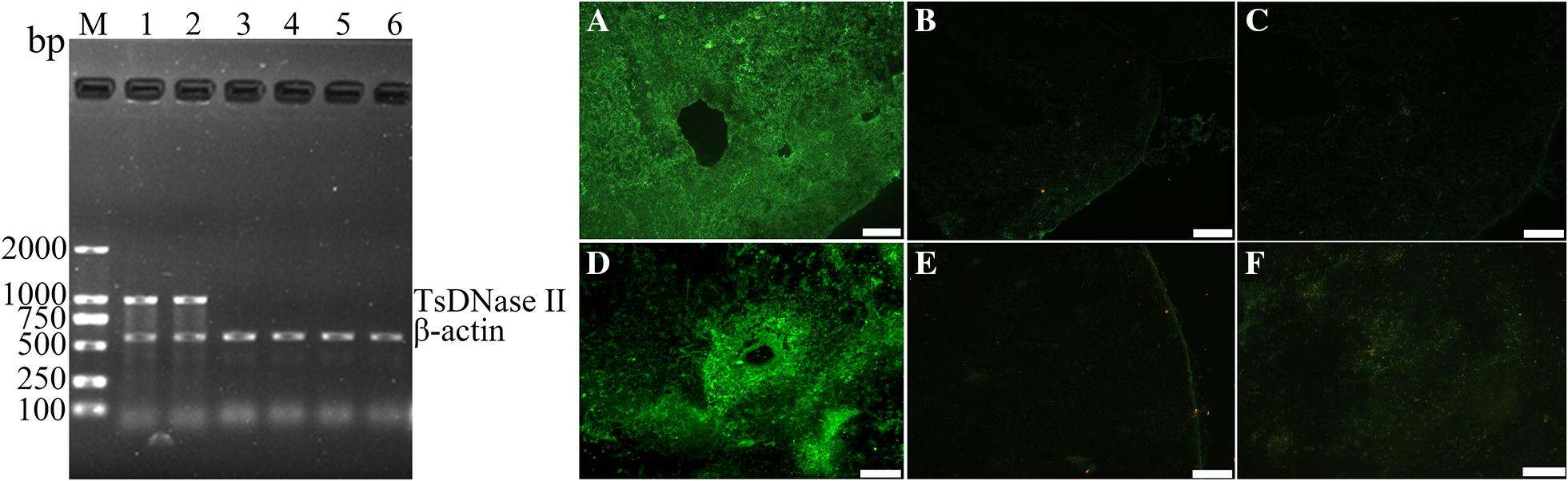
**Transcription and expression of recombinant pcDNA3.1-TsDNase II in the MLN and spleen of vaccinated mice.** Left: Transcriptions of TsDNase II in spleen and MLN of vaccinated mice were analyzed by RT-PCR. TsDNase II amplified fragments were observed in spleen (lane 1) and MLN (lane 2) from TsDNase II-vaccinated mice, but neither in spleen (lane 3) and MLN (lane 4) from pcDNA3.1 control mice, nor in spleen (lane 5) and MLN (lane 6) from the PBS group. Right: IIFT with anti-TsDNase II serum show that the fluorescence staining was detected in MLN (**A**) and spleen (**D**) of vaccinated mice, but the section of MLN (**B**) and spleen (**E**) of vaccinated mice were probed by normal serum. No immunostaining in MLN (**C**) and spleen (**F**) of pcDNA3.1 control mice was detected by anti-rTsDNase II serum. Scale bar: 50 μm.

Total RNA was extracted from mouse MLN and spleen at 1 week after the first immunization, the TsDNase II transcription in these tissues was determined using RT-PCR. The results reveal that the TsDNase II mRNA was detected in tissues of mice vaccinated with TsDNase II DNA, but not in the mice receiving only empty pcDNA3.1 or PBS (Figure 2). IIFT with anti-TsDNase II serum show that the fluorescence staining was detected in MLN and spleen from immunized mice with TsDNase II DNA vaccine. No staining was observed in MLN and spleen of mice inoculated with pcDNA3.1 alone. In addition, no fluorescence was seen in MLN and spleen tissues of immunized mice when IIFT with pre-immune serum was carried out (Figure 2). The results indicate that TsDNase II was expressed in MLN and spleen of mice vaccinated with TsDNase II DNA vaccine.

### Systemic humoral immune responses

**Figure 3:**
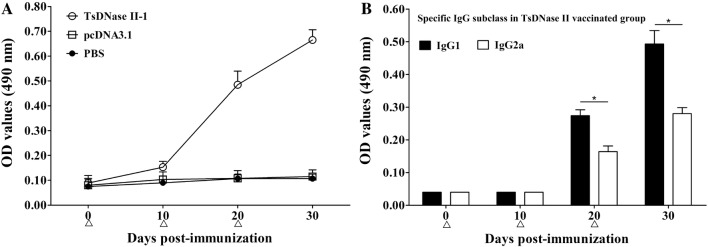
**Serum anti-TsDNase II IgG (A) and its subclass (B) responses in vaccinated mice were determined by ELISA with rTsDNase II.** OD values are the mean ± SD of antibody levels of ten animals from each group. Vaccination time is marked with triangles, and an asterisk represents significant differences (*P* < 0.01).

Serum samples collected from immunized mice were applied to determine the levels of TsDNase II-specific antibody IgG, IgG1 and IgG2a. Total specific IgG levels of mice vaccinated by TsDNase II vaccine was obviously raised at 10 days after the second and third vaccination (*F* = 491 308, *P *< 0.01) (Figure 3A). However, the mice inoculated only with pcDNA3.1 or PBS did not show overtly increased specific IgG levels. In TsDNase II vaccine-vaccinated mice, the IgG1 level on the 20^th^ and 30^th^ day after first vaccination was distinctly higher than IgG2a (*t*_20d_ = 14 097, *t*_30d_ = 15 057, *P *< 0.01) (Figure 3B). Nevertheless, the IgG2a was also triggered after the second vaccination with TsDNase II vaccines, suggesting that vaccination with TsDNase II DNA vaccines produced the mixed Th1/Th2 immune response with Th2 predominance.

### Intestinal mucosal immune response

**Figure 4:**
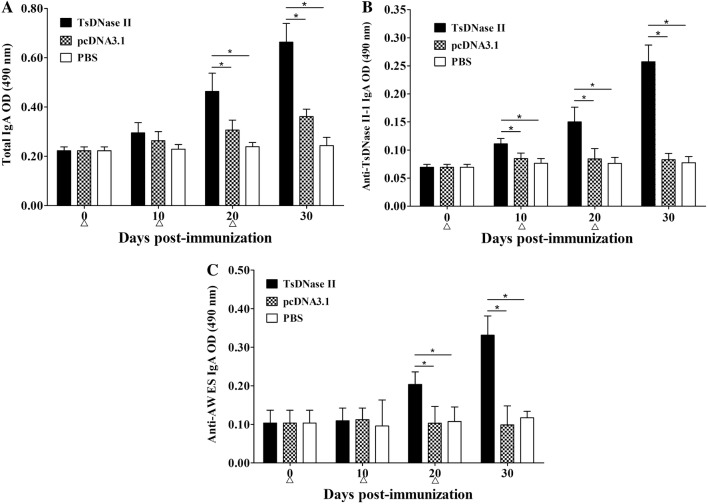
**Total intestinal IgA (A) of vaccinated mice assayed by sandwich ELISA and specific sIgA assayed by indirect ELISA using rTsDNase II (B) and AW ES antigens (C).** The results are the mean ± SD of 5 mice of each group, and asterisk indicates significant differences (*P* < 0.01).

To determine intestinal mucosal sIgA response to oral vaccination with TsDNase II DNA vaccine, total and TsDNase II-specific sIgA in intestinal washings were measured by ELISA (Figure 4). Both total and specific sIgA levels were prominently elevated after the second and third vaccination. On day 30 after vaccination, total sIgA level of TsDNase II-vaccinated mice was evidently higher than those of only pcDNA3.1 or the PBS group (*F* = 91 782, *P*_(TsDNase II, pcDNA3.1)_ < 0.01; *P*_(TsDNase II, PBS)_ < 0.01). TsDNase II-specific sIgA levels in TsDNase II-vaccinated mice were also distinctly higher than those of pcDNA3.1 alone or the PBS group (*F* = 139 526, *P*_(TsDNase II, pcDNA3.1)_ < 0.01; *P*_(TsDNase II, PBS)_ < 0.01). Moreover, when AW ES proteins were used as ELISA coating antigens, specific sIgA levels of TsDNase II-vaccinated mice were prominently higher than those of pcDNA3.1 alone or the PBS group (*F* = 44 403, *P*_(TsDNase II, pcDNA3.1)_ < 0.01; *P*_(TsDNase II, PBS)_ < 0.01).

### Recognition of the natural TsDNase II in different worm stages by IIFT

**Figure 5:**
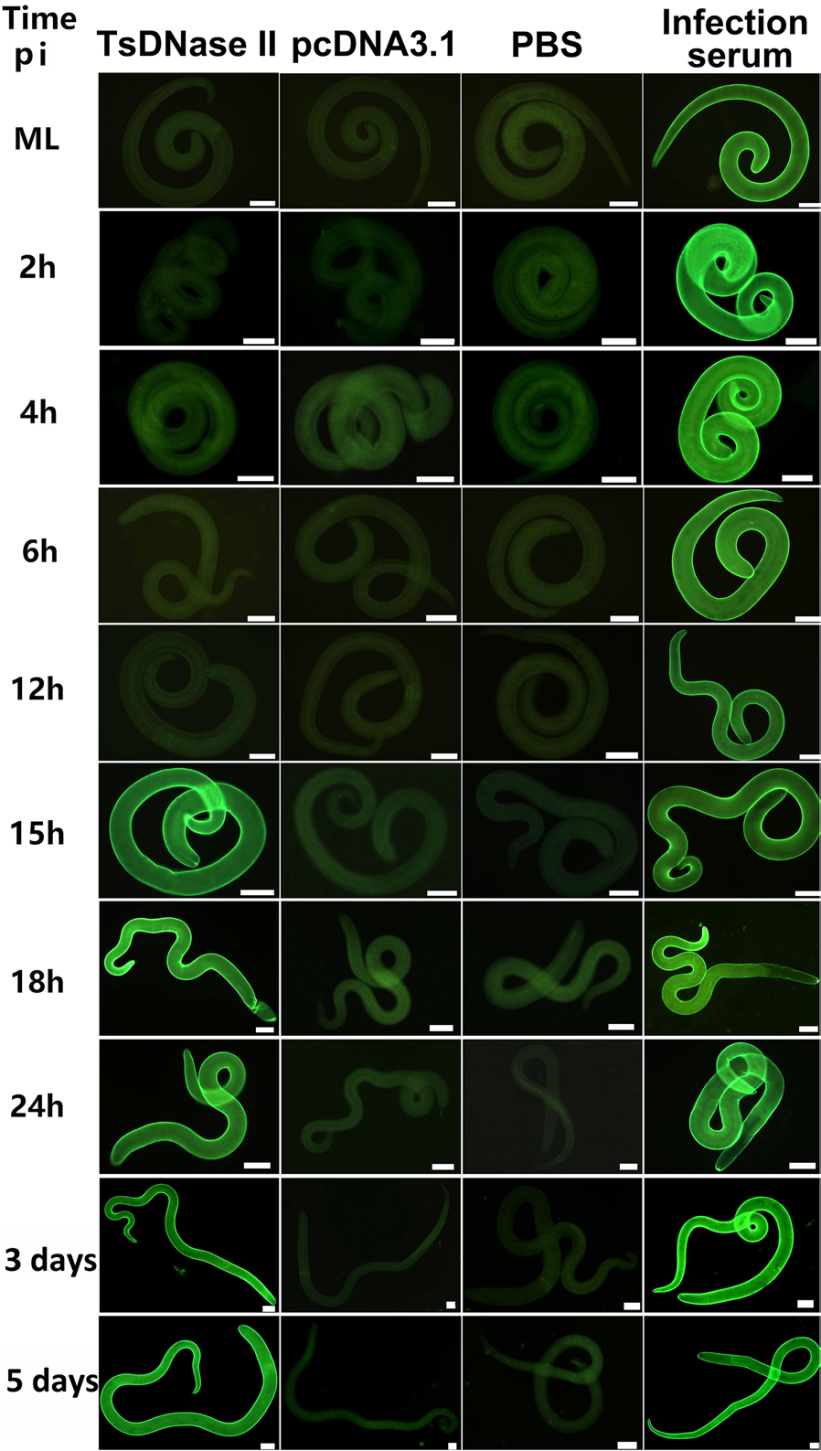
**Recognition of native TsDNase II at the cuticle of various T. spiralis phases by IIFT with serum from mice vaccinated with TsDNase II vaccine, pcDNA3.1 alone or PBS.** The intensive fluorescence staining at the external cuticle of IIL at 15, 18 and 24 hpi and adult worms at 3 and 5 dpi was detected by using anti-TsDNase II immune serum, but not by serum from only pcDNA3.1 or PBS control mice. No staining was seen on the cuticle of the ML and IIL at 2, 4, 6, 12 hpi by using anti-TsDNase II immune serum. The results indicate that native TsDNase II is present at the worm cuticle only after the 2^nd^ molting of this nematode. Besides, immunostaining was observed at the cuticle of all various *T. spiralis* phases using infection serum as positive control. Scale bar = 50 μm.

**Figure 6:**
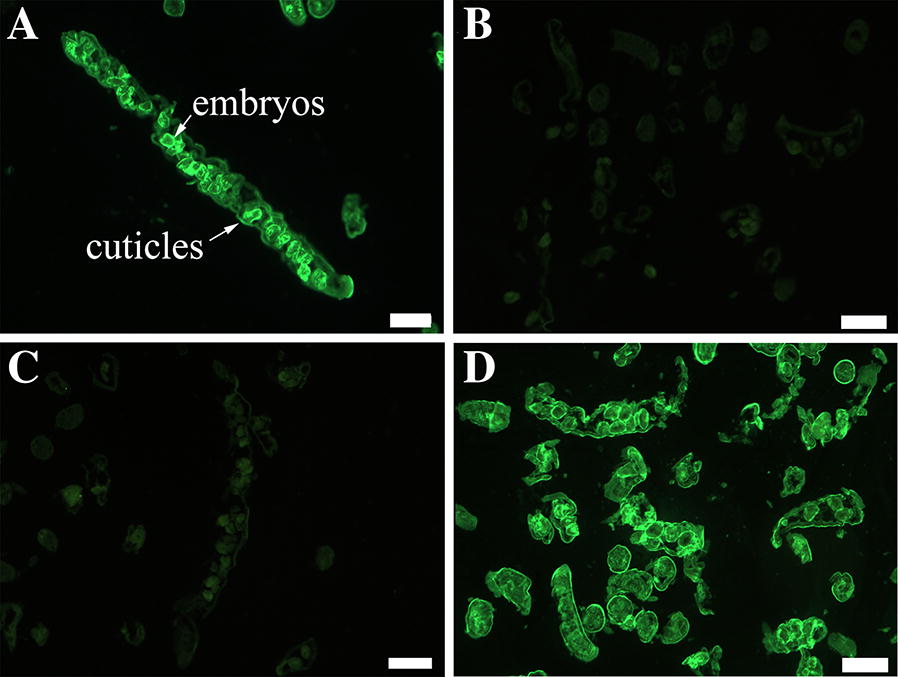
**Localization of natural TsDNase II in *T. spiralis* adult worms at 3 dpi by IIFT with serum of mice vaccinated with TsDNase II vaccine (A), pcDNA3.1 alone (B) or PBS (C)**. Infection serum was utilized as a positive control (**D**). Scale bar = 50 μm.

IIFT with anti-TsDNase II immune serum from mice vaccinated orally with TsDNase II DNA vaccine reveal that intensive fluorescence staining was observed on the external cuticle of IIL at 15, 18 and 24 hpi and adult worms at 3 and 5 dpi, but the staining was not detected using serum from only pcDNA3.1 or PBS control mice. Besides, no staining was seen on the cuticle of the ML and IIL at 2, 4, 6, 12 hpi by anti-TsDNase II immune serum (Figure 5). The results indicate that native TsDNase II is present in the worm cuticle only after the 2^nd^ molting of this nematode, further confirming that TsDNase II is adult-specific and expressed at *T. spiralis* adult and pre-adult stages. When adult sections were used, the staining was detected at the cuticle and embryos in 3 days adult female uteri (Figure 6). No immunostaining was found in the integral nematode or tissue sections probed by the serum from mice inoculated with only pcDNA3.1 or PBS. Additionally, native TsDNase II was also detected on the surface of 3 and 6 day-old *T. spiralis* AW, and NBL probed by intestinal anti-TsDNase II sIgA from immunized mice (Additional file 1). However, different *T. spiralis* stage worms were not stained by intestinal washings from mice inoculated with only pcDNA3.1 or PBS.

### Cytokine responses to the immunization

**Figure 7:**
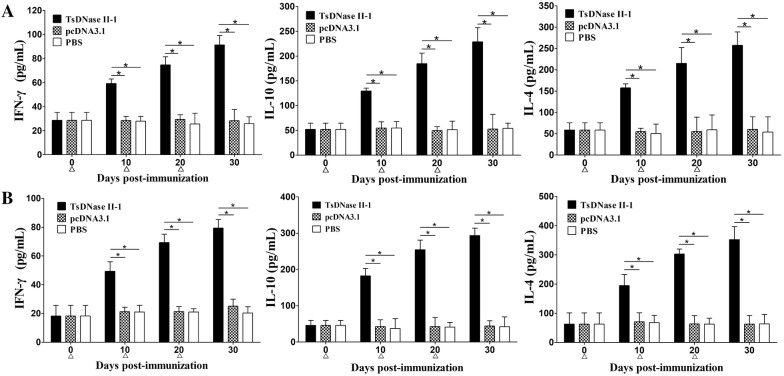
**IFN-γ, IL-10, and IL-4 upon rTsDNase II stimulation were assayed by ELISA. Concentration of cytokines in spleen (A) and MLN (B) cell supernatant was detected following stimulation with rTsDNase II for 72 h.** Data is presented as mean ± SD of cytokine concentrations (*n* = 5). Asterisk represents an evident difference between two groups (*P *< 0.01).

To investigate the cytokine production induced by rTsDNase II immunizations, spleen and MLN cells were collected from vaccinated and rTsDNase II stimulated mice. IFN-γ, IL-4, and IL-10 levels were assayed by sandwich ELISA. Obviously elevated levels of IFN-γ, IL-4 and IL-10 were observed in spleen and MLN cells from rTsDNase II-vaccinated mice on days 10, 20 and 30 after first vaccination compared to pcDNA3.1 and the PBS group (*P *< 0.01) (Figure 7). The results demonstrate that oral vaccination of mice with rTsDNase II significantly induced concurrent Th1/Th2 responses; it also suggests that the vaccination elicited systemically (spleen) and locally (MLN) immune response.

### Immune protection

**Figure 8:**
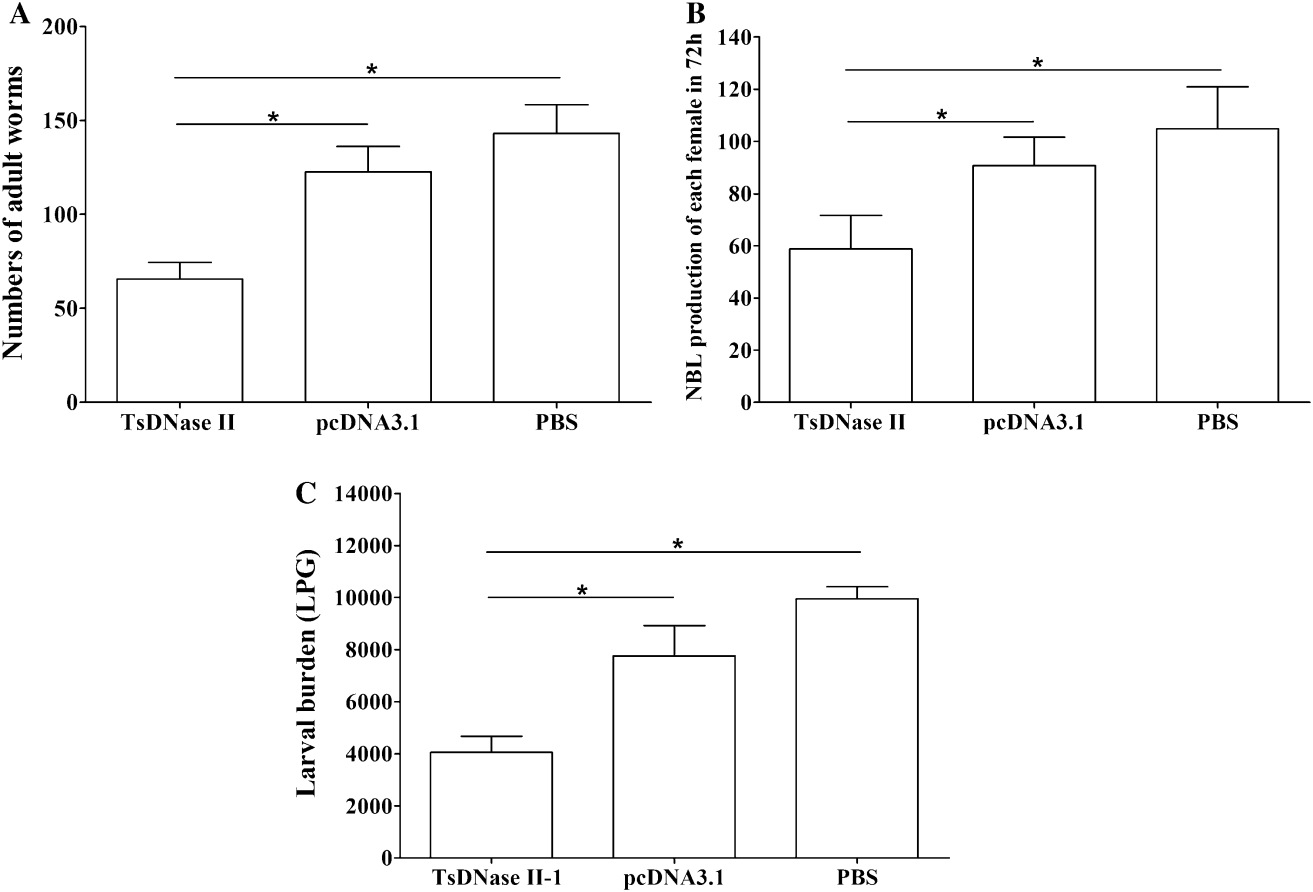
**Immune protection of mice orally immunized with TsDNase II DNA vaccine after challenge infection with 300 *T. spiralis* muscle larvae. A** Intestinal adult worms;** B** The in vitro NBL production of each female adult at 72 h;** C** Larvae per gram (LPG) of muscles. The worm numbers are shown as the mean ± SD from TsDNase II-immunized mice, pcDNA 3.1 and PBS control mice (*n* = 10). The significant difference was *P < 0.001.

Vaccination of mice with TsDNase II DNA vaccine shows a 53.85% of AW reduction compared to the PBS group, whereas the AW reduction of the pcDNA3.1 group was only 13.98%. The AW burden of TsDNase II-vaccinated mice was significantly lower than those of pcDNA3.1 and the PBS group (*F* = 97 401, *P*_(TsDNase II, pcDNA3.1)_ < 0.01; *P*_(TsDNase II, PBS)_ < 0.01) (Figure 8A). The in vitro NBL production of adult females from TsDNase II-vaccinated mice was also evidently reduced in comparison with pcDNA3.1 or the PBS control group (*F* = 15 385, *P*_(TsDNase II, pcDNA3.1)_ < 0.01; *P*_(TsDNase II, PBS)_ < 0.01) (Figure 8B). Vaccination of mice with TsDNase II DNA vaccine and pcDNA3.1 alone produced a 59.26% and 22.02% reduction of ML burden, respectively. The larval burden of TsDNase II-vaccinated mice was distinctly lower than that of pcDNA3.1 and the PBS group (*F* = 134 579, *P*_(TsDNase II, pcDNA3.1)_ < 0.01; *P*_(TsDNase II, PBS)_ < 0.01) (Figure 8C).

## Discussion

DNase enzymes play an active role in pathogen invading and immune evasion [51, 52]. A *Streptococcus* nuclease degrades extracellular chromatin in the form of neutrophil extracellular traps, and thereby helps it escape the host’s innate immune responses [53]. *Plasmodium* TatD-like DNase, which is involved in virulence and growth of the parasite, hydrolyzes the macrophage-derived extracellular trap structures in vitro; immunization of mice with rTatD-like DNase exhibited a parasitaemia reduction and delayed death [54]. *T. spiralis* DNase II has been identified in surface and ES proteins of different worm phases (AW, ML and IIL) [18, 20, 55, 56]. Our previous study revealed that subcutaneous vaccination with rTsDNase II induces high levels of TsDNase II-specific antibodies, which remarkably suppresses worm invasion of enterocytes in vitro, suggesting that DNase II may participate in the parasite-host interaction in early enteral stages of *T. spiralis* infection and could be served as a candidate target for anti-*Trichinella* vaccine [21].

To eliminate intestinal parasites from the gut, the immune response elicited by vaccination should disintegrate or expel the worms from the gut [57]. As the hosts acquire *Trichinella* infection through eating contaminated meat, oral vaccination with a DNA vaccine will be more appropriate to induce lasting intestinal mucosa protective responses to the enteral stage worms of *Trichinella* [8, 10]. In this study, a complete TsDNase II sequence was cloned into pcDNA3.1, and successfully expressed in BHK-21 cells in vitro. Recombinant pcDNA3.1/TsDNase II was transformed into attenuated *S. typhimurium* ⊿cyaSL1344 to produce a live bacteria-delivered TsDNase II DNA vaccine. Oral vaccination of mice with TsDNase II shows a prominent immune protection after larval challenge. Although the ML burden reduction (50.43%) of mice immunized orally with TsDNase II DNA vaccine is not statistically different from 59.26% of mice immunized subcutaneously with rTsDNase protein, intestinal AW burden reduction (53.85%) of mice immunized with TsDNase II DNA is significantly higher than 40.36% of mice immunized with rTsDNase [21]. The protection observed in this study is similar to that in mice immunized with the DNA vaccines of other *T. spiralis* proteins (nudix hydrolase, paramyosin) [40, 46], but lower than that of muscular injection with *T. spiralis* NBL serine protease (Ts-NBLsp) DNA vaccine; mice immunized with Ts-NBLsp DNA vaccine exhibited a 77·93% reduction in muscle larva burden after larval challenge [49]. This high protection might be due to the fact that NBL has the weakest resistance to the host’s immune response among various worm stages of *T. spiralis* lifecycle [58]. DNA vaccine with eukaryotic expression vector could improve the fold of recombinant protein and enable the post-translational modification, such as glycosylation. As a result, the surface-exposed epitopes of recombinant protein can be presented correctly as the structure of the native protein [40]. Live attenuated *Salmonella* has been proven as an effective vaccine vector which can deliver antigens to intestinal mucosa and lymphoid tissue and elicit long lasting systemic and mucosal protective responses to the enteral stage parasites [59]. Oral immunization with *Salmonella*-delivered *Trichinella* DNA vaccines displayed obvious reductions of AW and ML burdens in vaccinated mice [29, 35]. Our results reveal that TsDNase II mRNA and rTsDNase II protein in spleens and MLN of vaccinated mice are observed by RT-PCR and IIFT, demonstrating that TsDNase II was expressed in mouse tissues after being inoculated orally with TsDNase II DNA. Oral vaccination with TsDNase II DNA trigged significant systemic immune responses. Serum IgG1 level was obviously higher than IgG2a at 20 and 30 days following vaccination, but specific IgG2a was also produced after the 2^nd^ vaccination. This suggests that vaccination with TsDNase II DNA elicited the concurrent Th1/Th2 immune response with a Th2 predominance; this was certified by the elevated levels of Th1 (IFN-γ) and Th2 cytokines (IL-4 and IL-10) after MLN and spleen cells of vaccinated mice were stimulated with rTsDNase II. The mixed Th1/Th2 response is essential to protect the host from *Trichinella* infection [14, 28, 60].

Oral inoculation with TsDNase II DNA vaccine also elicited an evident intestinal local mucosal sIgA response. The sIgA has a principal effect in mucosal defense and can interrupt worm invasion of the intestinal epithelium. The sIgA against *T. spiralis* AW surface proteins mediated the worm expulsion of intestinal adults, passive transfer of IgA against *Trichinella* impeded establishment of *Trichinella* infective stage in intestinal mucosa of mice [61]. Our results indicate that vaccinated mice produced TsDNase II-specific IgG and intestinal sIgA, which recognized the natural TsDNase II at the cuticle of *T. spiralis* AW and pre-adult stages. Intestinal worm expulsion might be due to the formation of anti-*Trichinella* antibody immune complex in the worm head, which can physically interrupt the nematode invasion of enteral mucosa [62–64]. Furthermore, intestinal local sIgA inhibit the fecundity of adult females, and interrupt the parasite settlement in the intramulticellular niche of the intestinal epithelium [14, 65]. Our results show that the in vitro NBL production of females from TsDNase II-immunized mice was dramatically declined in comparison with pcDNA3.1 or PBS control mice, suggesting that TsDNase II-specific antibodies might inhibit intestinal worm development and reduce female fecundity [28, 66].

Successful preventive vaccines should be able to elicit strong immune responses that are long-lasting and provide high protection against different parasite stages. The efficient anti-*Trichinella* vaccine is supposed to control the enteral stage of the nematode, by facilitating AW expulsion from the gut and impeding NBL production and their migration to the skeletal muscle tissues [8]. However, the host’s immunity during *Trichinella* infection is usually stage-specific to various developmental stage worms [67]. In this study, since TsDNase II is a *Trichinella* adult-specific antigen that did not overlap with ML and IIL at 2–12 hpi, anti-TsDNase II antibodies (serum IgG and intestinal sIgA) recognized only the natural TsDNase II at the cuticle surface of AW and pre-adult stages at 15–24 hpi (as shown in Figure 6 and Additional file 1). These types of antibodies are unlikely to participate in worm rejection at early IIL stage and damaging of the ML stage mediated by antibody-dependent cellular cytotoxicity (ADCC) [68], therefore these antibodies might be the non-protective host antibody responses to early IIL and ML. As a consequence, immunization with adult-specific TsDNase II vaccine did not induce high protective immune response to other worm stages in vaccinated mice. *T. spiralis* is an intracellular parasite and has complicated lifecycle stages and stage-specific antigens. Vaccination with single recombinant adult-specific antigen molecules may not induce high protective responses in vaccinated mice, and is not enough to disable and dislodge the *Trichinella* parasites from the gut [8]. Therefore, in order to improve vaccination efficacy, the *Trichinella* common antigens in ES products and worm surface antigens from different worm stages are required to be used for development of anti-*Trichinella* vaccine, and the multivalent vaccines against various *T. spiralis* stages (especially the early IIL and NBL stages) should be explored [9]. Moreover, other vaccination strategies including the heterologous prime-boost immunization, genetic adjuvants or codon optimization, and different vaccination routes should also be developed [69]. For example, intranasal vaccination of mice with attenuated *Salmonella* expressing a *T. spiralis* gp43 antigen-derived 30-mer peptide fused with adjuvant C3d-P28 has achieved a 92.8% intestinal adult reduction following challenge infection [14].

In conclusion, our results indicate that a systemic concurrent Th1/Th2 immune response and intestinal mucosal sIgA response were triggered by oral inoculation with TsDNase II DNA delivered by attenuated *Salmonella* in a mouse model. The vaccinated mice produced a significant protective immunity, exhibited a 53.85% reduction of intestinal adults and a 59.26% reduction of muscle larvae after challenge. Oral vaccination with TsDNase II DNA provides a prospective strategy for the control of domestic animal *Trichinella* infection. Because *T. spiralis* is a multicellular intestinal- and tissue-dwelling parasitic nematode and has a complicated lifecycle and stage-specific antigenicity, polyvalent oral vaccines against different worm stages, especially the IIL, AW and NBL stages, need to be further developed.

## Additional file

**Additional file 1. Recognition of the native TsDNase II on the cuticle of *T. spiralis* various phase worms by IFT with intestinal sIgA of mice vaccinated with TsDNase II vaccine, pcDNA3.1 alone or PBS.** Immunostaining was detected on the surface of 3 and 6 day adults and NBL probed with intestinal washings from TsDNase II-immunized mice, but not by intestinal washings from pcDNA3.1 or PBS control mice. The surface of the ML and IIL was not recognized by intestinal washings from TsDNase II-immunized mice. Scale bar: 50 μm.

## Abbreviations

ADCC: antibody-dependent cellular cytotoxicity
AW: adult worms
BHK: baby hamster kidney cells
dpi: days post infection
ES: excretory/secretory
FBS: fetal bovine serum
IEC: intestinal epithelial cell
IIFT: indirect immunofluorescent test
IIL: intestinal infective larvae
LPG: larvae per gram
ML: muscle larvae
MLN: mesenteric lymph nodes
NBL: newborn larvae
OD: optical density
OPD: *o*-phenylenediamine dihydrochloride
SD: standard deviation
sIgA: secretory IgA
TsDNase II: *Trichinella spiralis* adult-specific DNase II-1
