# Supplementary figures and images for: Oral vaccination with Trichinella spiralis DNase II DNA vaccine delivered by attenuated Salmonella induces a protective immunity in BALB/c mice
Source: Vet Res. 2018 Dec 5;49:119. doi: 10.1186/s13567-018-0614-y (PMC6280372; doi:10.1186/s13567-018-0614-y)

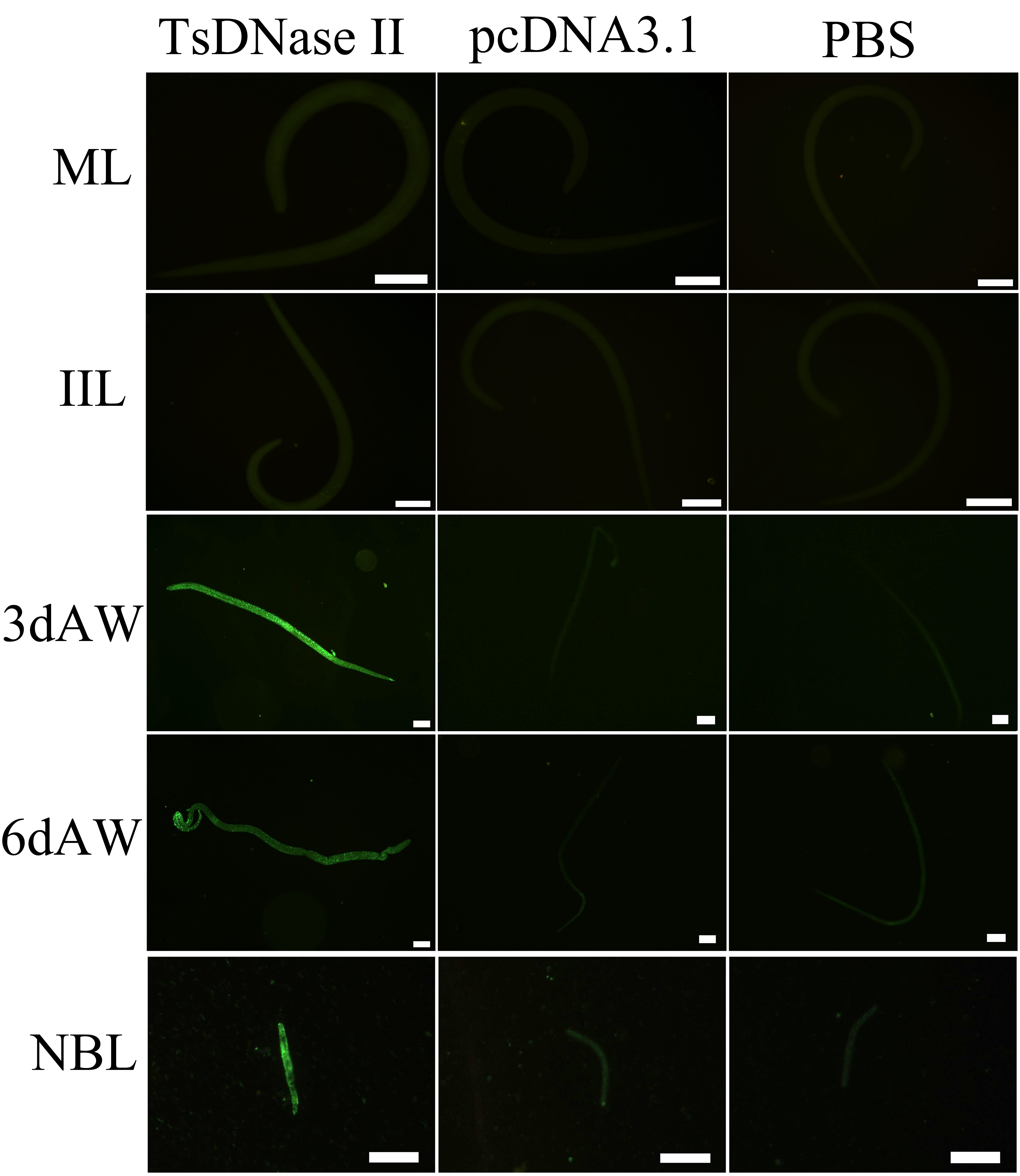

Supplement: Supplementary file 1 — Additional file 1. Recognition of the native TsDNase II on the cuticle of T. spiralis various phase worms by IFT with intestinal sIgA of mice vaccinated with TsDNase II vaccine, pcDNA3.1 alone or PBS. Immunostaining was detected on the surface of 3 and 6 day adults and NBL probed with intestinal washings from TsDNase II-immunized mice, but not by intestinal washings from pcDNA3.1 or PBS control mice. The surface of the ML and IIL was not recognized by intestinal washings from TsDNase II-immunized mice. Scale bar: 50 μm. [file 13567_2018_614_MOESM1_ESM.tif]
